# Supplementary material for: Identification of Crucial Genes and Regulatory Pathways in Alfalfa against Fusarium Root Rot
Source: Plants (Basel). 2023 Oct 21;12(20):3634. doi: 10.3390/plants12203634 (PMC10610399; doi:10.3390/plants12203634)
Supplement: Supplementary file 1 [file plants-12-03634-s001.zip › plants-2563063-SI.pdf]

Supplementary Material

Supplementary Table S1

Serial Number	Gene Id	Primer	Sequence（5′→3′）
1	MsG0580028496.01	1-2F	GATTCCGTTTGGTTCTGGGC
		1-2R	AGCTCCTTCAGGCATTTCCC
2	MsG0480022079.01	2-1F	CTGGCACAGACAAGGCAATC
		2-1R	AGTTTTTGACACCCTTGGTGG
3	MsG0280011264.01	3-1F	TCACACCAATGACGCAAGGA
		3-1R	AGGGAGCTCCAATTGATGGC
4	MsG0580028497.01	4-1F	AGACCCAGAAGCATGGAAGG
		4-1R	TTCGTCCAGAACCAAACGGA
5	MsG0880044995.01	5-1F	AGTTGCCTGAGGAGAAACGG
		5-1R	AATTCGCCATAGCCCACTCC
6	MsG0080048712.01	6-1F	AGTGTCACATGGCGTCTCTT
		6-1R	GTTCCACCCATGTCATTGCC
7	MsG0280010711.01	7-1F	TGGGGAAAATGGGTTGCTGA
		7-1R	ACTTAAACGCCTCGCGATCA
8	MsG0780040711.01	8-2F	CGCGCTAAGCGAGAGTATGA
		8-2R	AGTCCCCTTTTTCACCACCG
9	MsG0180004956.01	9-1F	AATCTTCGCCGGCAATTCTTG
		9-1R	CTCCGAGGCTGCGGATTATG
10	MsG0780041774.01	10-1F	GTTGGGTTGCATAGAGGTCC
		10-1R	GCCCAGCTCTTTTTGGAAGTG
11	MsG0280011105.01	11-1F	AATGTTTGACCGCAACGACG
		11-1R	CCACACATCCATCTCGGTTCA
12	MsG0380015289.01	12-2F	CGGTGGAGATACGAAGGAGC
		12-2R	AGCATTTCTCCCCACAACCG
13	MsG0480021441.01	13-2F	TTTGCTGTTTGGCGTGGAAG
		13-2R	TCGGCTACATGAGCGACATC
14	MsG0480021442.01	14-2F	ACACTCGCGATGGAAGTTCA
		14-2R	ACGTTGCAAGGTTTATGGCA
15	MsG0580025278.01	15-1F	AACTTCGTTCCGGCGACTTA
		15-1R	CGCCATCACCGTTTCAACTT
16	β-Actin	actin-F	TTCCCCGGCATTGCTGATAG
		actin-F	GGAGCCTCCAATCCAGACAC

Supplementary Table S2

Sample	Raw Data		Valid Data		Valid Ratio (reads)	Q20%	Q30%	GC content%
	Read	Base	Read	Base				
TMSR0-1	53351904	8.00G	51139572	7.67G	95.85	99.98	98.64	43
TMSR0-2	50174422	7.53G	47419192	7.11G	94.51	99.98	98.58	43
TMSR0-3	52924752	7.94G	50294942	7.54G	95.03	99.99	98.64	43
TMSR2-1	51936792	7.79G	45789964	6.87G	88.16	99.98	98.57	44
TMSR2-2	54790248	8.22G	48751414	7.31G	88.98	99.98	98.56	45
TMSR2-3	49235432	7.39G	45589510	6.84G	92.59	99.99	98.64	43
TMSR3-1	51617928	7.74G	48364274	7.25G	93.70	99.99	98.68	43
TMSR3-2	48395024	7.26G	45867894	6.88G	94.78	99.99	98.65	43
TMSR3-3	55419402	8.31G	51441560	7.72G	92.82	99.98	98.57	44
TMSS0-1	51333708	7.70G	48780436	7.32G	95.03	99.98	98.51	43
TMSS0-2	51769540	7.77G	49588546	7.44G	95.79	99.99	98.61	43
TMSS0-3	52189892	7.83G	49747756	7.46G	95.32	99.98	98.54	43
TMSS2-1	52871826	7.93G	50644682	7.60G	95.79	99.98	98.52	43
TMSS2-2	54685124	8.20G	51991892	7.80G	95.08	99.98	98.50	43
TMSS2-3	50675668	7.60G	48233210	7.23G	95.18	99.99	98.56	43
TMSS3-1	54920434	8.24G	50225292	7.53G	91.45	99.99	98.64	42
TMSS3-2	53221384	7.98G	50496290	7.57G	94.88	99.99	98.60	43
TMSS3-3	36398586	5.46G	34369922	5.16G	94.43	99.99	98.57	43
